# Toward understanding transcriptional regulatory networks in abiotic stress responses and tolerance in rice

**DOI:** 10.1186/1939-8433-5-6

**Published:** 2012-03-08

**Authors:** Daisuke Todaka, Kazuo Nakashima, Kazuo Shinozaki, Kazuko Yamaguchi-Shinozaki

**Affiliations:** 1 Biological Resources and Post-harvest Division, Japan International Research Center for Agricultural Sciences, Tsukuba, Ibaraki 305-8686, Japan; 2 RIKEN Plant Science Center, Yokohama, Kanagawa 230-0045, Japan; 3 Laboratory of Plant Molecular Physiology, Graduate School of Agricultural and Life Sciences, The University of Tokyo, Bunkyo-ku, Tokyo 113-8657, Japan

## Abstract

Abiotic stress causes loss of crop production. Under abiotic stress conditions, expression of many genes is induced, and their products have important roles in stress responses and tolerance. Progress has been made in understanding the biological roles of regulons in abiotic stress responses in rice. A number of transcription factors (TFs) regulate stress-responsive gene expression. OsDREB1s and OsDREB2s were identified as abiotic-stress responsive TFs that belong to the AP2/ERF family. Similar to *Arabidopsis*, these DREB regulons were most likely not involved in the abscisic acid (ABA) pathway. OsAREBs such as OsAREB1 were identified as key components in ABA-dependent transcriptional networks in rice. OsNAC/SNACs including OsNAC6 were characterized as factors that regulate expression of genes important for abiotic stress responses in rice. Here, we review on the rice abiotic-stress responses mediated by transcriptional networks, with the main focus on TFs that function in abiotic stress responses and confer stress tolerance in rice.

## Inroduction

In most crops, actual yields are only 20% of attainable yields (Boyer 1982). In crop production, a dominant factor limiting yield is abiotic stress such as excess or deficient water, high or low temperature, and high salinity (Boyer 1982). Therefore, improvement of abiotic stress tolerance might increase actual yields extensively in the most crops. As plants are sessile by nature they have evolved adaptive mechanisms against abiotic stress conditions. Recent progress in molecular biology has opened the door to uncovering the adaptive mechanisms at the molecular level in plants (Yamaguchi-Shinozaki & Shinozaki 2005, 2006).

A large number of abiotic stress responsive genes have been reported in a variety of plants including rice and *Arabidopsis*. These genes induced during stress conditions function not only in the protection of cells from stress by production of important metabolic proteins, but also in the regulation of genes, including transcription factors (TFs), for signal transduction in the stress responses. These TFs regulate expression of multiple downstream target genes under stress conditions. These regulatory systems are achieved through specific *cis*-elements in the promoter regions of target genes, which are termed 'regulons'. Several regulons involved in abiotic stress responses have been identified in *Arabidopsis *(Nakashima et al. 2009; Qin et al. 2011). The dehydration-responsive element binding protein 1 (DREB1)/C-repeat binding factor (CBF) regulon functions in the cold stress response, whereas the DREB2 regulon acts in heat and osmotic stress responses (Mizoi et al. 2011). The abscisic acid (ABA) responsive element (ABRE) binding protein (AREB)/ABRE binding factor (ABF) regulon functions in ABA-dependent gene expression under osmotic stress conditions (Fujita et al. 2011). In addition, the NAC regulon is shown to be involved in osmotic stress responses (Nakashima et al. 2009).

Rice (*Oryza sativa *L.) is a staple crop for more than half of the world's population. In addition to this social importance, rice shows promise as an excellent model plant for studies on cereals. The rice genome is one-sixth the size of the maize genome and 40 times smaller than the wheat genome (IRGSP 2005). The small genome size has enriched a set of resources available for molecular biological studies (Jung et al. 2008). Rice serves also as a model plant for studies of biofuel perennial grasses, such as switchgrass and *Miscanthus *(Kellogg 2001). Transcriptomic analyses of rice treated with abiotic stresses have been carried out (Cooper et al. 2003; Hazen et al. 2005; Lan et al. 2005; Rabbani et al. 2003; Ray et al. 2011; Wang et al. 2007; Zhou et al. 2007). These analyses clarified a large number of stress-responsive genes, which can be divided into two groups. One group is a signaling component that regulates gene expression in the stress responses, including protein kinases and TFs. The other group is a functional component that directly protects plant cells against the stress, including enzymes in metabolic pathways, aquaporins, and late embryogenesis abundant (LEA) proteins. Efforts to identify and characterize these stress-responsive genes have uncovered several abiotic stress regulatory networks in rice. Here, we review the rice abiotic stress responses mediated by transcriptional networks, and focus on TFs that function in abiotic stress responses and confer stress tolerance in rice.

### Overview of abiotic stress responses in rice

**Figure 1:**
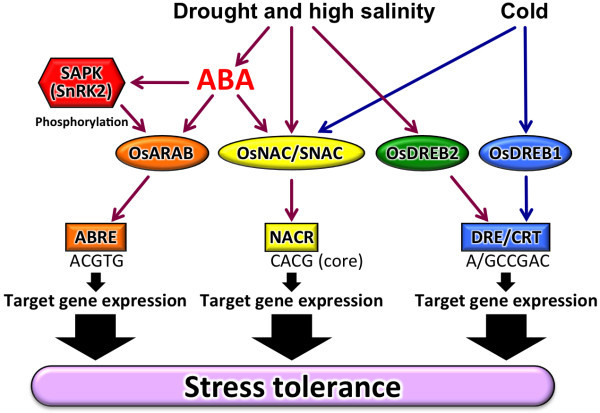
**Overview of the representative transcriptional networks mediated by transcription factors and *cis*-elements under abiotic stresses in rice**. Ellipses represent transcription factors, boxes represent *cis*-elements.

Numerous stress-responsive genes have been identified in transcriptomic analyses of rice treated with abiotic stresses Rabbani et al. (2003). identified 36, 62, and 57 genes that were induced by cold, drought, and high salinity, respectively. These included genes encoding calmodulin, catalase, LEA protein, metallothionein-like protein, transcription factors such as zinc finger, NAC, and Myb, lipoxygenase, and sugar transporter protein Wang et al. (2007). compared comprehensive expression profiles between genotypes of upland rice, which is resistant to water stress, and lowland rice, which is susceptible to water stress, that were subjected to PEG treatment. The authors found 13% of genes expressed in leaves and 7% of genes expressed in roots showed differential expression between the two genotypes. Genes highly expressed in upland rice included TFs, proteins conferring detoxification or protection against oxidative stress, and proteins that maintain cell turgor. Genes highly expressed in lowland rice were involved in the degradation of cellular components. Zhou et al. (2007) examined expression patterns under drought or high salinity stress in the shoot, flag leaf, and panicle. The majority of genes expressed in response to the stress treatments were organ-specific. For example, the number of genes expressed specifically in the drought-treated shoot, flag leaf, and panicle were 1020, 301, and 448, respectively, whereas the number of genes expressed in all of these organs was 42. The latter genes included protein kinases, chlorophyll a/b binding protein, LEA proteins, and transcription factors such as zinc finger type and homeobox type. Thus, expression of thousands of genes is regulated by a variety of transcriptional cascades when rice plants are subjected to abiotic stresses. Transcriptomic analysis of other cereal crops, including barley (Oztur et al. 2002; Talame et al. 2007), maize (Andjelkovic & Thompson 2006; Luo et al. 2010; Yu & Setter 2003; Zheng et al. 2004) and sorghum (Buchanan et al. 2005), has shown that thousands of genes are up- or down-regulated under abiotic stress conditions. Therefore, analysis of transcriptional regulatory systems is important to clarify abiotic stress responses and tolerance. Some regulons, such as DREB, AREB, and NAC, have been analyzed extensively in rice (Figure 1) and *Arabidopsis*. Evidence is emerging that similar, but not identical, regulons exist in rice compared to *Arabidopsis*. Regulons modulated by DREB1, DREB2, AREB, NAC, and other TFs, and other stress-responsive genes in rice are reviewed in the following sections. TFs described in this paper are listed in Table 1.

**Table 1:** A list of transcription factors (TFs) described in this paper

Gene name	Tolerance in OX (mutant)	Phenomena or roles in stress response	Reference
OsDREB1 TFs			
OsDREB1A	Drought ↑, Salt ↑, Cold ↑	Accumulation of proline and regulation of stress-responsive gene expression	(Ito et al. 2006)
OsDREB1B	Drought ↑, Salt ↑, Cold ↑	Induction of the TF expression in cold stress	(Ito et al. 2006)
OsDREB1F	Drought ↑, Salt ↑, Cold ↑	Regulation of ABA-responsive gene expression	(Wang et al. 2008)
OsDREB1G	Drought ↑	Binding to the DRE element	(Chen et al. 2008)
OsDREB2 TFs			
OsDREB2A	Not reported	Induction of the TF expression in heat, drought and salt stresses	(Matsukura et al. 2010)
OsDREB2B	Drought ↑, Heat ↑	Regulation of gene expression via stress-induced alternative splicing	(Matsukura et al. 2010)
OsAREB TFs			
TRAB1	Not reported	Regulation of gene expression via phosphorylation in response to ABA	(Hobo et al. 1999; Kagaya et al. 2002)
OsABF2	Drought ↓, Salt ↓, Cold ↓	Modulation of gene expression through an ABA-dependent pathway	(Hossain et al. 2010)
OsABI5	Salt ↓, (Salt ↑)	Regulation of adaptive stress response and plant fertility	(Zou et al. 2008)
OsbZIP23	Drought ↑, Salt ↑	Regulation of expression of genes involved in stress response and tolerance	(Xiang et al. 2008)
ABL1	Not reported	Modulation of ABA and auxin responses	(Yang et al. 2011)
OsbZIP72	Drought ↑	Regulation of ABA responsive gene expression	(Lu et al. 2009)
OsNAC TFs			
OsNAC6/SNAC2	Drought ↑, Salt ↑	Regulation of abiotic and biotic stress responsive gene expression	(Nakashima et al. 2007; Hu et al. 2008)
OsNAC5	Salt ↑	Regulation of OsLEA3 expression	(Takasaki et al. 2010; Song et al. 2011)
SNAC1	Drought ↑	Increase of stomatal closure and expression of genes involved in stress tolerance	(Hu et al. 2006)
OsNAC10	Drought ↑	Enhancement of grain yield under drought conditions	(Jeong et al. 2010)
Other TFs			
AP37	Drought ↑	Enhancement of grain yield	(Oh et al. 2009)
AP59	Drought ↑	Spikelet disruption	(Oh et al. 2009)
ZFP252	Drought ↑, Salt ↑	Accumulation of proline and sugars	(Xu et al. 2008)
ZFP245	Drought ↑, Cold ↑	Elevation of ROS enzyme activity	(Huang et al. 2009a)
ZFP179	Salt ↑	Accumulation of proline and sugars	(Sun et al. 2010)
DST	(Drought ↑, Salt)	Regulation H202-induced stomatal closure	(Huang et al. 2009b)
OsWRKY45-2	Salt ↓ (Salt ↑)	Regulation of ABA sensitivity	(Tao et al. 2009)
OsTIFY11a	Salt ↑, Mannitol ↑	Regulation seed germination	(Ye et al. 2009)
Osmyb4	Chilling ↑	The TF decreases membrane injury	(Vannini et al. 2004; Park et al. 2010)
OsMYB3R-2	Drought ↑, Salt ↑, Cold ↑	Regulation of G2/M phase specific gene expression	(Dai et al. 2007)

### DREB1/CBF regulon

*Arabidopsis *DREB1-type genes *DREB1A, DREB1B *and *DREB1C *were identified as transcription factors that bind to a *cis*-acting element, DRE/CRT. The DRE/CRT contains the core sequence A/GCCGAC important for regulation of gene expression in response to drought, high salinity, and cold stresses in *Arabidopsis *(Yamaguchi-Shinozaki & Shinozaki 2005). The expression of *DREB1A, DREB1B *and *DREB1C *was induced by cold stress and the three encoded DREB1 proteins were major transcriptional activators required for cold-inducible gene expression. Transgenic *Arabidopsis *overexpressing DREB1-type genes driven by the *Cauliflower mosaic virus *(CaMV) 35S promoter displayed strong tolerance to drought, high salinity, and freezing with growth retardation (Yamaguchi-Shinozaki & Shinozaki 2006). Use of a stress-inducible promoter minimized the growth retardation. More than 100 downstream target genes were identified and the most of these genes possessed the DRE core motif in their promoter regions (Yamaguchi-Shinozaki & Shinozaki 2006).

The rice genome contains at least ten DREB1-type genes, among which *OsDREB1A *and *OsDREB1B *are induced by cold stress (Dubouzet et al. 2003). Overexpression of *OsDREB1A *induced strong expression of stress-responsive genes in transgenic *Arabidopsis *plants, which resulted in not only improved stress tolerance to high-salinity and freezing stresses, but also growth retardation of plants under non-stress growth conditions (Dubouzet et al. 2003). This result indicates OsDREB1A shows functional similarity to DREB1A. In addition, transgenic rice overexpressing *OsDREB1A *and *OsDREB1B *also showed growth retardation under non-stress conditions and improved tolerance to drought, high-salinity and cold stress (Ito et al. 2006). The transgenic rice plants accumulated osmoprotectants, including proline and a variety of sugars, under control conditions as occurred in the transgenic *Arabidopsis *(Ito et al. 2006). Target stress-inducible genes of OsDREB1A were identified by microarray analysis and encode proteins thought to function in stress tolerance in the plants, which is consistent with the target genes of DREB1 proteins in *Arabidopsis*. These observations indicate the DREB1 regulon is conserved in rice and DREB1-type genes are useful for improvement of tolerance to environmental stresses in transgenic plants including rice.

*OsDREB1F *is induced by salt, drought, and cold stresses, and also ABA application (Wang et al. 2008). Transgenic rice plants harboring the *OsDREB1F *gene showed enhanced stress tolerance to salt, drought, and cold stress. Overexpression of *OsDREB1F *in *Arabidopsis *enhanced expression of stress-inducible genes, such as *rd29B *and *RAB18*, that contain the DRE/CRT element in their promoter regions, which suggests that OsDREB1F might also participate in expression of osmotic stress responsive genes through the ABA-dependent signaling pathway. Overexpression of *OsDREB1G *also elevates drought tolerance (Chen et al. 2008). These results indicate the DREB1 regulon in rice is one of the master regulatory system in abiotic stress responses.

### DREB2 regulon

The *Arabidopsis *DREB2 protein also has a conserved ERF/AP2 DNA-binding domain and recognizes the DRE sequence. However, the expression pattern and transcriptional regulatory mechanism of DREB2 differed from those of DREB1. *DREB2A *and *DREB2B *were induced by drought, high salinity and high temperature. The encoded DREB2 proteins function as major transcriptional activators required for expression of genes inducible by these stresses. Overexpression of *DREB2A *in transgenic *Arabidopsis *neither caused growth retardation nor improved stress tolerance (Yamaguchi-Shinozaki & Shinozaki 2006). A negative regulatory domain was indicated to exist in the central region of DREB2A and deletion of this region transforms DREB2A into a constitutively active form (*DREB2A CA*) (Qin et al. 2011; Mizoi et al. 2011). Overexpression of *DREB2A CA *in transgenic *Arabidopsis *increased expression of many stress-inducible genes and resulted in enhanced tolerance to drought, salt and heat stresses (Mizoi et al. 2011). The protein stability mediated by ubiquitin E3 ligases is important for activation of DREB2A (Qin et al. 2011).

Rice has at least four *DREB2 *homologues, among which *OsDREB2A *and *OsDREB2B *are induced by drought, high salinity and heat stresses (Matsukura et al. 2010). *OsDREB2B *is also induced by cold stress. *OsDREB2B *expression is regulated by alternative splicing that generates two-types of transcripts, namely functional and nonfunctional forms. The functional transcript encodes the full-length protein, whereas the nonfunctional form has a stop codon within the full-length protein. Although the nonfunctional form of transcripts accumulates under non-stress conditions, the functional form of transcripts is induced by abiotic stresses. The alternative splicing of *DREB2*-type genes was observed also in other monocotyledonous plants such as barley (Xue & Loveridge 2004), wheat (Egawa et al. 2006) and maize (Qin et al. 2007). Transgenic *Arabidopsis *plants overexpressing the functional form of *OsDREB2B *displayed enhanced expression of DREB2A target genes and improved tolerance to drought and heat stresses (Matsukura et al. 2010). These analyses indicate *OsDREB2B *is a key gene that encodes the DREB2-type TF that functions in stress-responsive gene expression in rice. Transgenic *Arabidopsis *plants overexpressing the functional form of maize *ZmDREB2A *showed improved tolerance to drought and heat stresses (Qin et al. 2007). Microarray analysis showed that the up-regulated genes in *Arabidopsis *plants overexpressing *ZmDREB2A *were similar to those in *Arabidopsis *plants overexpressing *DREB2A CA *(Qin et al. 2007; Sakuma et al. 2006). Collectively, the function of *DREB2*-type genes in different plants is similar but the regulatory systems of *DREB2*-type gene expression are most likely different between monocotyledons and dicotyledons; the former requires RNA processing, whereas the latter changes protein stability.

### AREB regulon

Abscisic Acid acts as a crucial signal molecule in abiotic stress responses (Fujita et al. 2011). The ABA content is increased by abiotic stresses, and leads to expression of numerous genes. Application of exogenous ABA also stimulates a myriad of genes. ABRE was identified as a cis-acting element conserved in promoter regions of ABA-inducible genes. *Arabidopsis *cDNAs that encode bZIP-type TFs were screened as ABRE-binding proteins (Yamaguchi-Shinozaki & Shinozaki 2006). Among these genes, *AREB1/ABF2, AREB2/ABF4*, and *ABF3 *were reported to be induced by ABA and osmotic stress in vegetative tissues (Fujita et al. 2011). Evidence indicates that activation of AREB1 needs ABA-dependent posttranscriptional modification. The ABA-activated SnRK2 protein kinases phosphorylate the AREB1 protein (Furihata et al. 2006). Transgenic *Arabidopsis *plants overexpressing the phosphorylated active form of AREB1 showed enhanced expression of a number of ABA-inducible genes (Furihata et al. 2006). The ABA-activated phosphorylation of AREB/ABFs was completely impaired in the SnRK2 triple mutant, *srk2d srk2e srk2i *(Fujii et al. 2009; Fujii & Zhu 2009). The down-regulated genes in the *srk2d srk2e srk2i *and *areb1 areb2 abf3 *triple mutants largely overlapped in ABA-dependent expression, which supports the view that SRK2D/E/I regulate AREBs in ABA signaling in response to osmotic stress. (Fujita et al. 2009).

The rice TRANSCRIPTION FACTOR RESPONSIBLE FOR ABA REGULATION1 (TRAB1) shows high homology to *Arabidopsis *AREB2/ABF4. Expression of *TRAB1 *was up-regulated by ABA treatment (Hobo et al. 1999). TRAB1 is phosphorylated rapidly in response to ABA treatment (Kagaya et al. 2002). One of the rice bZIP-type TFs, *OsABF2*, was shown to be induced by drought, high salinity, cold and oxidative stresses, and ABA treatment (Hossain et al. 2010). A T-DNA insertion mutant of *OsABF2 *showed increased sensitivity to abiotic stresses compared to wild-type plants. The loss of function approach suggests that OsABF2 positively functions in abiotic stress signaling. Another rice bZIP-type TF, OsABI5, was isolated from rice panicles (Zou et al. 2008). Expression of *OsABI5 *was induced by ABA and high salinity, but it was down-regulated by drought and cold stresses in rice seedlings. Transgenic rice plants overexpressing *OsABI5 *showed high sensitivity to salt stress. In contrast, antisense *OsABI5 *transgenic rice plants showed increased stress tolerance but decreased fertility (Zou et al. 2008). These results indicate OsABI5 might regulate adaptation to stress and plant fertility. (Nijhawan et al. 2008) surveyed the rice genome for bZIP family proteins and analyzed the expression of 89 *OsbZIP *genes. Their microarray analysis indicated 26 genes were up-regulated and 11 genes were down-regulated under dehydration, salinity, and/or cold conditions.

The function of several abiotic stress-inducible rice bZIP-type TFs has been studied in transgenic rice plants and identification of their downstream genes has been attempted by microarray analyses. Expression of *OsbZIP23 *was induced by drought, high salinity and ABA (Xiang et al. 2008). Transgenic rice overexpressing *OsbZIP23 *exhibited improved tolerance to drought and high-salinity stresses. In contrast, a null mutant for this gene showed decreased tolerance to high-salinity and drought stress. Microarray analyses revealed 37 possible OsbZIP23-specific target genes, which showed changes of reverse expression level in the overexpressor and mutant. *ABI5-Like1 *(*ABL1*) was expressed in a number of tissues and its expression was induced by ABA and indole-3-acetic acid treatments, and by drought and salinity stresses (Yang et al. 2011). The *abl1 *mutant showed suppressed ABA responses. Microarray analysis showed that a large proportion of down-regulated genes in the *abl1 *mutant are involved in stress responses. ABL1 also regulated a series of ABRE-containing WRKY family genes. In addition, the *abl1 *mutant showed hypersensitivity to exogenous indole-3-acetic acid, and altered expression of genes related to auxin metabolism or signaling. These mutant analyses suggest that ABL1 modulates ABA and auxin responses. Transgenic rice plants overexpressing *OsbZIP72 *showed hypersensitivity to ABA, elevated levels of expression of ABA-responsive genes such as *LEAs*, and enhanced drought tolerance (Lu et al. 2009). These observations suggest rice AREB homologues regulate ABA-inducible gene expression, similar to the functions of *Arabidopsis *AREB proteins.

Ten SnRK2 protein kinases have been characterized in rice (Kobayashi et al. 2004). All family members are activated by hyperosmotic stress. Three genes of the family were also activated by ABA and these ABA-activated SnRK2 proteins can phosphorylate TRAB1 (Kobayashi et al. 2005). OSRK1 was isolated and encodes a 41.8-kD protein kinase of the SnRK2 family (Chae et al. 2007). *In vitro *kinase assays demonstrated that OSRK1 can phosphorylate both itself and generic substrates. OREB1, a rice ABRE-binding factor, was phosphorylated in vitro by OSRK1 at multiple sites of different functional domains. Ectopic expression of OSRK1 in transgenic tobacco caused reduced sensitivity to ABA in seed germination and root elongation. These findings indicate OSRK1 is associated with ABA signaling, possibly through the phosphorylation of the ABF family *in vivo*. Collectively, these findings suggest the ABA-activated SnRK2 protein kinases phosphorylate and activate the AREB/ABF-type proteins in rice, which leads to the enhanced expression of a number of genes involved in abiotic stress responses and tolerance.

### NAC regulon

NAC-type transcription factors that regulate expression of abiotic stress responsive genes were isolated initially from *Arabidopsis*. In *Arabidopsis*, three NACs (ANAC019, ANAC055, and ANAC072) were isolated as TFs that regulate expression of a salt- and drought-induced gene, *ERD1 *(Yamaguchi-Shinozaki & Shinozaki 2006). Expression of the three *NAC *genes was enhanced under salt and drought stress conditions. Microarray analysis of transgenic *Arabidopsis *plants overexpressing these NAC genes showed several stress-inducible genes were up-regulated in the transgenic plants. These up-regulated genes caused significantly increased tolerance to drought in the transgenic lines (Yamaguchi-Shinozaki & Shinozaki 2006).

In rice several *NAC *genes are reported to be induced by drought, high salinity, and cold stresses. *OsNAC6/SNAC2 *encodes a NAC transcription factor in rice (Hu et al. 2008; Nakashima et al. 2007). Expression of OsNAC6/SNAC2 is induced by cold, drought and high salinity and also is enhanced by jasmonic acid, wounding and blast disease. Transgenic rice plants overexpressing *OsNAC6 *showed enhanced tolerance to dehydration and high salinity stresses, although the plants showed growth retardation and low yield under the non-stress condition. These negative effects were minimized in transgenic rice plants driven by a stress-inducible promoter. Microarray analyses using the *OsNAC6 *overexpressors clarified the downstream genes, which included many stress-inducible genes. Among these genes, two genes, one of which was a gene that encoded peroxidase, were activated in a transient transactivation assay. These results indicate OsNAC6 functions as a transcriptional activator in both abiotic and biotic stress responses. Furthermore, a rice gene that encodes a histone deacetylase, *OsHDAC1*, epigenetically represses *OsNAC6 *expression (Chung et al. 2009).

*OsNAC5 *expression was induced by dehydration, cold, ABA, and MeJA treatments (Takasaki et al. 2010). The growth of rice plants overexpressing *OsNAC5 *was similar to that of control plants under non-stress conditions. The *OsNAC5 *overexpressors showed improved tolerance to high salinity. Microarray analyses using the *OsNAC5 *overexpressors identified downstream genes, which included *OsLEA3*. OsNAC5 was shown to bind the NAC recognition core sequence (CACG) in the OsLEA3 promoter region in a gel mobility shift assay. Song et al. (2011) reported that RNA interference (RNAi) transgenic rice plants with reduced *OsNAC5 *expression showed less tolerance to abiotic stresses than control plants, whereas overexpression of *OsNAC5 *enhanced abiotic stress tolerance. It was also shown that accumulation of proline and soluble sugars was positively correlated with *OsNAC5 *expression level. SNAC1 was identified as a key component that functions in stress responses in rice (Hu et al. 2006). *SNAC1 *expression was induced by drought, salt, cold and ABA treatment. Overexpression of *SNAC1 *improved drought tolerance. The *SNAC1 *overexpressors did not show any negative effects on growth and productivity. Crystal structure analysis of the SNAC1 protein revealed the overall architecture and two loop regions essential for DNA binding (Chen et al. 2011). *OsNAC10 *was expressed in roots and panicles under non-stress conditions (Jeong et al. 2010). The expression was induced by drought, high salinity, and ABA. Root-specific overexpression of *OsNAC10 *enhanced tolerance and grain yield in the field under drought conditions.

These results indicate that, in addition to the DREB and AREB regulons, the NAC regulon is also important for transcriptional networks of abiotic stress responses both in monocotyledons and dicotyledons.

### Other TF regulons

Other TFs are reported to act in abiotic stress responses in rice. These include AP2/ERFs (AP37, AP59), C_2_H_2 _zinc fingers (ZFP252, ZFP245, ZFP179 and DST), a WRKY (OsWRKY45), a TIFY (OsTIFY11a), and MYBs (Osmyb4, and OsMYB3R-2). Transgenic rice plants overexpressing *AP37 *or *AP59 *showed increased tolerance to drought (Oh et al. 2009). Overexpression of *AP37 *improved grain yield under drought conditions, but overexpression of *AP59 *caused spikelet disruption and resulted to lower yield than WT. Overexpression of *ZFP252*, a C_2_H_2_-type zinc finger protein, increased salt and drought stress tolerance, and elevated expression of stress-responsive genes such as *OsDREB1A *(Xu et al. 2008). Transgenic rice plants overexpressing *ZFP245 *showed significant tolerance to cold and drought stresses (Huang et al. 2009a). In the overexpressors, the activities of reactive oxygen species-scavenging enzymes were elevated under stress conditions. Overexpression of *ZFP179 *increased the levels of proline and soluble sugars, leading to enhanced salt tolerance (Sun et al. 2010). DST mutants displayed enhanced tolerance to drought and salt stresses, which was mediated by H_2_O_2_-induced stomatal closure (Huang et al. 2009b). *OsWRKY45-1 *and *OsWRKY45-2 *were isolated from *japonica *and *indica *rice, respectively (Tao et al. 2009). Expression of both genes was induced by cold stress, but was reduced under drought stress (Tao et al. 2011). Transgenic rice plants overexpressing *OsWRKY45-1 *showed reduced ABA sensitivity, whereas overexpressors of *OsWRKY45-2 *displayed increased ABA sensitivity and salt stress tolerance. Expression of the *OsTIFY11a *gene was induced by cold, drought and salt stresses (Ye et al. 2009). Overexpressors of *OsTIFY11a *showed improved salt and mannitol tolerance. Overexpression of *Osmyb4*, a cold-inducible MYB TF gene, enhanced cold and freezing tolerance in transgenic *Arabidopsis *plants (Vannini et al. 2004). Transgenic rice plants overexpressing *Osmyb4 *showed enhanced chilling tolerance (Park et al. 2010). The plants exhibited decreased membrane injury and a high germination rate under a low temperature. Transcriptome analysis using *Osmyb4 *overexpressors showed that expression of genes associated with cellular defense and rescue, metabolism and development were changed in the overexpressors. Challenges ectopically expressing *Osmyb4 *were performed in other crops such as apple (Pasquali et al. 2008) and *Osteospermum ecklonis *(Laura et al. 2010), an ornamental and perennial plant native to South Africa. These plants showed enhanced tolerance to cold and drought stresses through phenylpropanoid metabolism and biosynthesis of compatible osmolytes. OsMYB3R-2 was also reported to be a stress-inducible MYB TF (Dai et al. 2007). Transgenic *Arabidopsis *plants overexpressing OsMYB3R-2 showed elevated tolerance to cold, drought and high-salinity stresses with high expression levels of stress-inducible genes such as *DREB2A *and *COR15a*. These findings indicate that a variety of TFs function in abiotic stress responses and tolerance in rice. These TFs could be useful for development of transgenic crops with enhanced tolerance to abiotic stresses.

### Abiotic stress tolerance mechanisms of transgenic rice plants or mutants with altered TF expression

One of the mechanisms that contribute to abiotic stress tolerance is the accumulation of osmoprotectants in plant cells. Osmoprotectant compounds include amino acids, onium compounds and polyoles/sugars. These compounds are produced in cells subjected to abiotic stresses and stabilize proteins and cell membranes. Transgenic Arabidopsis plants overexpressing *DREB1A *showed up-regulated expression of genes that encode osmoprotectant biosynthesis proteins, which leads to the accumulation of osmoprotectants, such as myo-inositol, sucrose, galactinol and raffinose (Maruyama et al. 2009). This finding indicates that DREB1A functions as a regulator of osmoprotectant biosynthesis to enhance stress tolerance. Transgenic rice plants overexpressing *DREB1A *also accumulated Pro and sugars. Although a metabolic change in *OsDREB1A *overexpressors has not been identified successfully, it is suggested that *DREB1A *orthologues can function in the osmoprotectant biosynthesis pathways in rice similar to those in Arabidopsis.

LEA proteins are implicated in the mechanism of desiccation tolerance (Goyal et al. 2005). Overexpression of *OsLEA3-1 *in rice improved grain yield under drought conditions (Xiao et al. 2007). Some genes that encode LEA proteins, including OsLEA3-1, were up-regulated in *OsbZIP23 *overexpressors (Xiang et al. 2008). One mechanism that contributes to the enhanced tolerance in *OsbZIP23 *overexpressors may be the result of up-regulated *LEA *gene expression. *OsLEA3-1 *was also up-regulated in *OsNAC5 *overexpressors (Takasaki et al. 2010). These authors demonstrated that OsNAC5 and OsNAC6 can bind to the promoter region of *OsLEA3-1*.

The relationship among each TF in abiotic stress responses is not fully understood. We compared OsDREB1A, AP59, OsbZIP23, SNAC1, OsNAC5 and OsNAC6 downstream genes identified by microarray analyses. No downstream genes were up-regulated in all overexpressors of these TFs. Most downstream genes were specifically up-regulated in each overexpressor. For example, 72 downstream genes in *AP59 *overexpressors and 156 downstream genes in *OsNAC6 *overexpressors did not overlap, and only two genes, Os10g0419400 and Os01g0795200, which encode acireductone dioxygenase and subtilisin-like serine proteinase, respectively, overlapped. Only one gene, Os10g0450900, which encodes glycine-rich protein, was up-regulated in three overexpressors, namely *OsDREB1A, OsbZIP23 *and *OsNAC6 *overexpressors. These results suggest that OsDREB1A, AP59, OsbZIP23, SNAC1, OsNAC5 and OsNAC6 TFs function independently in abiotic stress responses with a specific set of downstream genes.

## Conclusions

Significant progress has been made in understanding the biological roles of several regulons in abiotic stress responses in rice. TFs play important roles in the signaling cascades. OsDREB1A, OsDREB1B, OsDREB1F, OsDREB2A, and OsDREB2B were identified as abiotic stress responsive DREB-type TFs in rice. Similar to *Arabidopsis*, these regulons were most likely not involved in the ABA signaling pathway. Several TFs were characterized that regulate expression of ABA-responsive genes in rice. OsABF2, OsbZIP23, ABL1, OsbZIP72, TRAB1, and OsABI5 are thought to function as key components in ABA-dependent transcriptional networks in rice. The NAC-type TFs OsNAC6, SNAC1, OsNAC5, and OsNAC10 are also important regulons in abiotic stress responses in rice.

Ray et al. (2011) reported that 1,563 and 1,746 genes were up- or down-regulated, respectively, under water-deficit conditions, respectively. Among these genes, members of zinc finger TFs such as C2C2, C3H, LIM, PHD, ZF-HD, and TFs that encode GeBP, jumonji, MBF1, and ULT were newly identified as TFs differentially expressed under water stress. The role of these TFs in the stress response remains unclear.

Development of genetically modulated rice plants that show enhanced tolerance and yield under adverse conditions is still a challenge. One key objective is to identify the genes responsible for abiotic stress tolerance in rice. Variations exist in tolerance mechanisms among plant species. Understanding the crosstalk between different stress signal transduction pathways is also important. Because plants are highly adaptable and have many mechanisms to survive environmental stresses, dissection of abiotic stress responses will be an important clue to enhance crop yield.

## Competing interests

The authors declare that they have no competing interests.

## Authors' contributions

DT wrote the paper, KN drew a figure, and KS and KY-S designed research. All authors read and approved the final manuscript.
